# *CTLA-4* polymorphisms and predisposition to digestive system malignancies: a meta-analysis of 31 published studies

**DOI:** 10.1186/s12957-020-1806-2

**Published:** 2020-03-16

**Authors:** Jien Li, Wenping Wang, Yanyan Sun, Yeduo Zhu

**Affiliations:** 1 Department of Oncology, Changyi People’s Hospital, Changyi, 261300 Shandong Province China; 2 Department of Gastroenterology, Changyi People’s Hospital, Changyi, 261300 Shandong Province China; 3 Department of Gastroenterology, Zhuji Affiliated Hospital of Shaoxing University, Zhuji, 311800 Zhejiang China

**Keywords:** Cytotoxic T lymphocyte-associated antigen 4 (CTLA-4), Polymorphisms, Digestive system malignancies, Meta-analysis

## Abstract

**Background:**

The results of genetic association studies regarding cytotoxic T lymphocyte-associated antigen 4 (*CTLA-4*) polymorphisms and digestive system malignancies were controversial. The authors designed this meta-analysis to more precisely estimate relationships between *CTLA-4* polymorphisms and digestive system malignancies by pooling the results of related studies.

**Methods:**

The authors searched PubMed, Embase, Web of Science, and CNKI for eligible studies. Thirty-one eligible studies were pooled analyzed in this meta-analysis.

**Results:**

The pooled meta-analysis results showed that genetic distributions of rs231775, rs4553808, and rs733618 polymorphisms among patients with digestive system malignancies and controls differed significantly. Moreover, genotypic distribution differences were also observed for rs231775 polymorphism among patients with colorectal cancer/pancreatic cancer and controls, for rs4553808 and rs5742909 polymorphisms among patients with gastric cancer and controls, for rs3087243 polymorphism among patients with liver cancer and controls, and for rs733618 polymorphism among patients with colorectal cancer and controls in pooled meta-analyses.

**Conclusions:**

This meta-analysis suggested that rs231775 polymorphism was associated with predisposition to colorectal cancer and pancreatic cancer, rs4553808 and rs5742909 polymorphisms were associated with predisposition to gastric cancer, rs3087243 polymorphism was associated with predisposition to liver cancer, and rs733618 polymorphism was associated with predisposition to colorectal cancer.

## Background

Digestive system malignancies such as liver cancer, gastric cancer, and colorectal cancer are leading types of cancer among both males and females [1, 2]. Although their definite pathogenesis mechanisms are still unclear, accumulating evidence suggests that genetic architecture plays vital roles in their development. Firstly, the incidences of digestive system malignancies have been found to be higher in subjects with positive family history in first-degree relatives [3–5], and genetic background is probably one of the reasons behind this phenomenon. Secondly, previous genetic association studies have also detected numerous susceptible genetic loci of digestive system malignancies in different populations [6–8]. However, the pathogenesis mechanisms of digestive system malignancies are very complicated, and genetic factors that contribute to the development of digestive system malignancies still require intensive explorations.

Cytotoxic T lymphocyte-associated antigen 4 (CTLA-4) serves as a negative regulator of immune responses and is essential for modulating anti-tumor immune responses [9, 10]. So, if a polymorphism is of potential functional significance and can impact the gene expression or protein structure of CTLA-4, it is likely that this polymorphism might also influence predisposition to many types of malignant diseases including digestive system malignancies.

In the last two decades, investigators across the world have extensively explored the relationship between *CTLA-4* polymorphisms and digestive system malignancies, yet the relationships between *CTLA-4* polymorphisms and digestive system malignancies are still controversial and ambiguous. Thus, the authors designed this meta-analysis to get a more statistically reliable conclusion regarding the relationships between *CTLA-4* polymorphisms and digestive system malignancies by pooling the results of related studies.

## Methods

The PRISMA guideline was followed by the authors when conducting this meta-analysis [11].

### Literature search and inclusion criteria

Literature searching of PubMed, Web of Science, Embase, and CNKI was performed by the authors using the following terms: (Cytotoxic T lymphocyte antigen-4 or CTLA-4) and (polymorphism or variant or variation or mutation or SNP or genome-wide association study or genetic association study or genotype or allele) and (colorectal or colon or rectal or pancreatic or pancreas or esophageal or esophagus or gastric or stomach or liver or hepatic) and (cancer or tumor or carcinoma or neoplasm or malignancy). The authors also checked the references of retrieved articles for additional related studies.

Eligible studies must meet all of the three inclusion criteria: (I) formally published case-control studies evaluating relationships between *CTLA-4* polymorphisms and digestive system malignancies, (II) provide genotypic distributions of *CTLA-4* polymorphisms in patients with digestive system malignancies and controls, and (III) the full manuscript is available in English or Chinese. Articles were excluded if at least one of the following three conditions was fulfilled: (I) studies not concerning *CTLA-4* polymorphisms and digestive system malignancies, (II) reviews or expert comments, and (III) case series that only involved patients with digestive system malignancies. When duplicate reports were observed during literature searching, only the most complete one was included for pooled analyses.

### Data extraction and quality assessment

We extracted the following items from eligible studies: (I) surname of the first author, (II) year of online publication, (III) country and ethnicity of involved subjects, (IV) number of patients and controls in each study, and (V) genotypic distributions of *CTLA-4* polymorphisms in patients and control subjects. We also calculated the *p* values of Hardy-Weinberg equilibrium (HWE) based on genotypic distributions of *CTLA-4* polymorphisms.

The authors used the Newcastle-Ottawa scale (NOS) to assess the quality of included studies [12]. The score range of NOS is from zero to nine, and the methodology quality of a study is considered to be good if it can get a score of more than seven.

Data extraction and quality assessment of eligible studies were performed by two authors separately. We would write to the corresponding authors of eligible studies for additional data if we fail to extract necessary information from included studies.

### Statistical analyses

The authors used Review Manager to pool the results of eligible studies. The authors used *Z* test to evaluate the relationships between *CTLA-4* polymorphisms and predisposition to digestive system malignancies. The authors set the statistical significant threshold at 0.05. We compared genetic distributions of *CTLA-4* polymorphisms among cases and controls in dominant, recessive, over-dominant, and allele models; the dominant genetic model is defined as M/M vs. M/m + m/m, recessive genetic model is defined as m/m vs. M/M + M/m, over-dominant genetic model is defined as M/m vs. M/M + m/m, and the allele genetic model is defined as M vs. m. The authors used *I*^2^ statistics to estimate heterogeneity. The authors used the DerSimonian-Laird method to pool the results if *I*^2^ is larger than 50%. Otherwise, the authors used the Mantel-Haenszel method to pool the results. The authors also conducted subgroup analyses by type of diseases. The authors examined the stabilities of pooled meta-analysis results by omitting one study each time and pooling the results of the other studies. The authors examined publication biases by using funnel plots.

## Results

### Characteristics of included studies

**Fig. 1:**
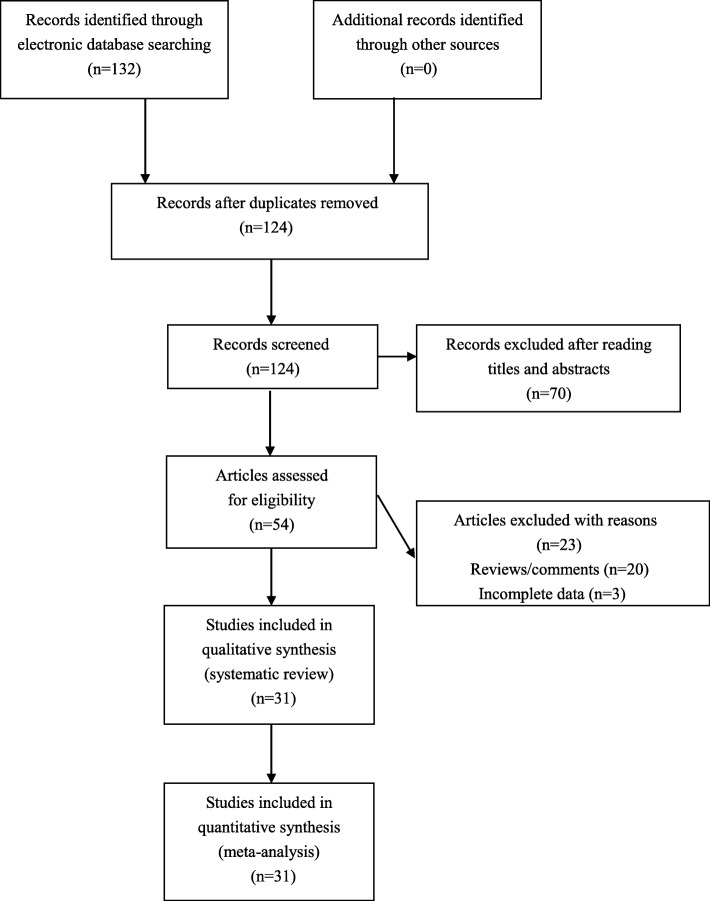
Flowchart of study selection for the present study

One hundred and thirty-two articles were retrieved by the authors through our literature searching strategy. The authors assessed 54 articles for eligibility after omitting unrelated and repeated reports. Twenty reviews were further excluded by the authors, and another three articles were also excluded by the authors due to the lack of crucial data. Totally, 31 studies were finally pooled in our meta-analyses (Fig. 1). Extracted data of eligible studies were summarized in Table 1.

**Table 1:** The characteristics of included studies for this meta-analysis

First author, year	Country	Ethnicity	Type of disease	Sample size	Genotypes (wtwt/wtmt/mtmt)		*P* value for HWE	NOS score
					Cases	Controls		
rs231775 A/G								
Cai 2011*	China	East Asian	Esophageal cancer	125/250	30/68/27	70/133/47	0.248	8
Cheng 2006*	Taiwan	East Asian	Gastric cancer	62/250	34/26/2	119/102/29	0.323	7
Cheng 2011*	China	East Asian	Esophageal cancer	205/205	54/105/46	90/79/36	0.013	7
Cozar 2007*	Spain	Caucasian	Colorectal cancer	96/176	46/44/6	78/77/21	0.766	8
Cui 2012*	China	East Asian	Colorectal cancer	128/205	73/46/9	84/68/53	< 0.001	7
Cui 2016*	China	East Asian	Liver cancer	96/205	52/37/7	64/96/45	0.429	7
Dilmec 2008*	Turkey	Mixed	Colorectal cancer	56/162	36/19/1	108/43/11	0.028	7
Fan 2012*	China	East Asian	Colorectal cancer	291/352	123/146/22	170/138/44	0.059	8
Ge 2015*	China	East Asian	Colorectal cancer	572/626	296/242/34	292/284/50	0.094	7
Gu 2010*	China	East Asian	Liver cancer	367/407	150/166/51	183/179/45	0.902	8
Hadinia 2007*	Iran	Mixed	Colorectal cancer	105/190	52/47/6	117/59/14	0.097	8
Hadinia 2007*	Iran	Mixed	Gastric cancer	46/190	27/13/6	117/59/14	0.097	8
Hou 2010*	China	East Asian	Gastric cancer	205/262	94/70/41	107/100/55	< 0.001	7
Hu 2010*	China	East Asian	Liver cancer	853/854	367/380/106	399/376/79	0.476	8
Lang 2012*	China	East Asian	Pancreatic cancer	602/651	208/312/82	263/326/62	0.006	8
Li 2011*	China	East Asian	Colorectal cancer	248/380	120/120/8	171/167/42	0.898	8
Liu 2015*	China	East Asian	Liver cancer	80/78	29/36/15	38/33/7	0.966	7
Liu 2015*	China	East Asian	Esophageal cancer	629/686	307/254/43	310/296/58	0.284	7
Liu 2019*	China	East Asian	Gastric cancer	487/1470	228/215/44	698/631/141	0.926	7
Mahajan 2008*	Poland	Caucasian	Gastric cancer	301/411	89/153/59	152/189/70	0.393	7
Qi 2010*	China	East Asian	Colorectal cancer	124/407	60/60/4	183/179/45	0.902	8
Solerio 2005*	Italy	Caucasian	Colorectal cancer	132/238	76/43/13	128/91/19	0.618	8
Sun 2008*	China	East Asian	Gastric cancer	530/530	235/235/60	282/209/39	0.974	8
Sun 2008*	China	East Asian	Esophageal cancer	629/686	307/254/43	310/290/58	0.398	8
Tang 2016*	China	East Asian	Gastric cancer	330/590	155/153/22	278/264/48	0.179	8
Wang 2015*	China	East Asian	Colorectal cancer	311/389	121/147/43	141/147/101	< 0.001	7
Yang 2012*	China	East Asian	Pancreatic cancer	368/926	140/178/50	482/374/70	0.828	8
Yang 2015*	China	East Asian	Colorectal cancer	240/147	195/39/6	102/40/5	0.662	7
Yang 2019*	China	East Asian	Liver cancer	575/920	290/221/64	444/389/87	0.893	8
Yuan 2012*	China	East Asian	Gastric cancer	118/96	65/45/8	30/45/21	0.595	7
Zou 2018*	China	East Asian	Colorectal cancer	979/1299	417/443/119	621/563/115	0.430	8
rs4553808 A/G								
Cui 2016*	China	East Asian	Liver cancer	96/205	33/56/7	79/96/30	0.924	7
Hadinia 2007*	Iran	Mixed	Colorectal cancer	109/188	74/33/2	145/36/7	0.02	7
Hadinia 2007*	Iran	Mixed	Gastric cancer	46/188	37/9/0	145/36/7	0.02	7
Hou 2010*	China	East Asian	Gastric cancer	205/262	112/71/22	163/54/45	< 0.001	7
rs5742909 C/T								
Cheng 2006*	Taiwan	East Asian	Gastric cancer	62/250	59/3/0	209/40/1	0.323	7
Dilmec 2008*	Turkey	Mixed	Colorectal cancer	56/162	48/8/0	149/12/1	0.185	7
Hadinia 2007*	Iran	Mixed	Gastric cancer	46/187	38/8/0	159/24/4	0.014	8
Hadinia 2007*	Iran	Mixed	Colorectal cancer	108/187	91/16/1	159/24/4	0.013	7
Li 2009*	China	East Asian	Gastric cancer	236/121	206/27/3	99/17/5	0.001	7
Wang 2019*	China	East Asian	Liver cancer	554/612	360/170/24	466/134/12	0.517	8
Yang 2015*	China	East Asian	Colorectal cancer	240/147	150/82/8	94/48/5	0.707	7
rs3087243 CT60AG								
Cheng 2006*	Taiwan	East Asian	Gastric cancer	62/250	39/20/3	154/79/17	0.126	7
Cozar 2007*	Spain	Caucasian	Colorectal cancer	95/175	20/56/19	40/88/47	0.923	7
Ge 2015*	China	East Asian	Colorectal cancer	1699/627	1258/425/16	413/198/16	0.174	8
Liu 2019*	China	East Asian	Gastric cancer	487/1472	302/172/13	958/462/52	0.686	8
Tang 2016*	China	East Asian	Gastric cancer	316/580	213/98/5	382/182/16	0.302	8
Wang 2019*	China	East Asian	Liver cancer	554/612	200/238/116	240/274/98	0.185	8
Yang 2019*	China	East Asian	Liver cancer	575/921	325/221/29	609/282/30	0.703	8
Zou 2018*	China	East Asian	Colorectal cancer	980/1300	637/296/47	850/408/42	0.410	8
rs733618 T/C								
Cui 2016*	China	East Asian	Liver cancer	96/205	72/24/0	181/24/0	0.373	7
Hadinia 2007*	Iran	Mixed	Colorectal cancer	109/189	97/12/0	165/24/0	0.351	7
Hadinia 2007*	Iran	Mixed	Gastric cancer	83/189	42/41/0	165/24/0	0.351	7
Hou 2010*	China	East Asian	Gastric cancer	205/262	75/111/19	93/139/30	0.041	7
Liu 2019*	China	East Asian	Gastric cancer	487/1472	168/242/77	525/685/262	0.139	8
Tang 2014*	China	East Asian	Esophageal cancer	611/657	210/300/101	228/314/115	0.700	8
Tang 2016*	China	East Asian	Gastric cancer	320/586	102/163/55	198/282/106	0.749	8
Yang 2019*	China	East Asian	Liver cancer	575/921	217/268/90	320/432/169	0.275	8
Zou 2018*	China	East Asian	Colorectal cancer	980/1300	346/464/170	458/613/229	0.335	8
rs16840252 C/T								
Liu 2019*	China	East Asian	Gastric cancer	492/1472	381/94/7	1130/329/13	0.039	8
Tang 2016*	China	East Asian	Gastric cancer	317/603	235/78/4	460/130/13	0.293	8
Yang 2019*	China	East Asian	Liver cancer	575/921	477/93/5	707/205/9	0.164	8
Zou 2018*	China	East Asian	Colorectal cancer	980/1300	742/223/15	1006/283/11	0.065	8

*Abbreviations*: *wt* wild type, *mt* mutant type, *HWE* Hardy-Weinberg equilibrium, *NOS* Newcastle-Ottawa scale, *NA* not available

*Full manuscript of all eligible studies can be accessed at https://osf.io

### Meta-analysis results of CTLA-4 polymorphisms and digestive system malignancies

Twenty-eight studies were eligible for estimation of relationship between rs231775 polymorphism and digestive system malignancies, three studies were eligible for estimation of relationship between rs4553808 polymorphism and digestive system malignancies, six studies were eligible for estimation of relationship between rs5742909 polymorphism and digestive system malignancies, eight studies were eligible for estimation of relationship between rs3087243 polymorphism and digestive system malignancies, eight studies were eligible for estimation of relationship between rs733618 polymorphism and digestive system malignancies, and four studies were eligible for estimation of relationship between rs16840252 polymorphism and digestive system malignancies. *CTLA-4* rs231775 (over-dominant comparison: OR = 1.06, *p* = 0.03), rs4553808 (dominant comparison: OR = 0.77, *p* = 0.04; recessive comparison: OR = 0.52, *p* = 0.003; over-dominant comparison: OR = 1.73, *p* < 0.0001), and rs733618 (over-dominant comparison: OR = 1.27, *p* = 0.04) polymorphisms were found to be significantly associated with digestive system malignancies in overall pooled meta-analyses. We also obtained positive findings for rs231775 polymorphism in colorectal cancer (recessive and over-dominant comparisons) and pancreatic cancer (dominant, recessive, over-dominant, and allele comparisons) subgroups, for rs4553808 polymorphism in gastric cancer (recessive and over-dominant comparisons) subgroup, for rs5742909 polymorphism in gastric cancer (dominant and allele comparisons) subgroup, for rs3087243 polymorphism in liver cancer (dominant, recessive, and allele comparisons) subgroup, and for rs733618 polymorphism in colorectal cancer (allele comparison) subgroup. Nevertheless, no any positive results were observed for rs16840252 polymorphism in pooled meta-analyses (see Table 2).

**Table 2:** Meta-analysis results of this study

Variables	Sample size	Dominant comparison		Recessive comparison		Over-dominant comparison		Allele comparison	
		M/M vs. M/m + m/m		m/m vs. M/M + M/m		M/m vs. M/M + m/m		M vs. m	
		*p* value	OR (95%CI)	*p* value	OR (95%CI)	*p* value	OR (95%CI)	*p* value	OR (95%CI)
rs231775									
Overall	9890/14,238	0.91	0.99 (0.89–1.11)	0.13	0.86 (0.71–1.05)	*0.03*	1.06 (1.00–1.12)	0.22	1.06 (0.96–1.17)
Esophageal cancer	1588/1827	0.47	0.87 (0.60–1.26)	0.71	0.96 (0.77–1.20)	0.67	1.05 (0.83–1.35)	0.57	0.93 (0.93–1.19)
Gastric cancer	2079/3799	0.84	1.02 (0.81–1.29)	0.65	0.92 (0.66–1.30)	0.27	1.07 (0.95–1.19)	0.16	1.14 (0.95–1.37)
Colorectal cancer	3042/4424	0.64	1.04 (0.88–1.23)	0.007	0.55 (0.35–0.84)	0.006	1.14 (1.04–1.25)	0.09	1.16 (0.98–1.38)
Liver cancer	1971/2464	0.82	1.04 (0.76–1.41)	0.57	1.13 (0.75–1.70)	0.41	0.95 (0.84–1.07)	0.98	1.00 (0.77–1.31)
Pancreatic cancer	970/1577	< 0.0001	0.67 (0.57–0.79)	0.0001	1.67 (1.29–2.16)	0.03	1.20 (1.02–1.42)	< 0.0001	0.73 (0.64–0.82)
rs4553808									
Overall	456/843	0.04	0.77 (0.60–0.98)	0.003	0.52 (0.34–0.81)	< 0.0001	1.73 (1.34–2.24)	0.87	0.98 (0.81–1.19)
Gastric cancer	251/450	0.20	0.80 (0.57–1.12)	0.03	0.56 (0.33–0.95)	0.002	1.77 (1.23–2.55)	0.86	1.02 (0.78–1.34)
rs5742909									
Overall	1302/1666	0.76	0.94 (0.63–1.40)	0.42	1.22 (0.75–1.99)	0.42	1.15 (0.82–1.62)	0.58	0.87 (0.54–1.41)
Gastric cancer	344/558	0.04	1.58 (1.01–2.48)	0.14	0.40 (0.12–1.36)	0.46	0.74 (0.33–1.66)	0.01	1.69 (1.12–2.56)
Colorectal cancer	404/496	0.45	0.88 (0.63–1.23)	0.66	0.81 (0.32–2.08)	0.33	1.19 (0.84–1.68)	0.15	0.58 (0.28–1.21)
rs3087243									
Overall	4768/5937	0.72	0.96 (0.79–1.17)	0.66	0.92 (0.64–1.33)	0.70	1.03 (0.87–1.23)	0.90	0.99 (0.84–1.17)
Gastric cancer	865/2302	0.53	0.95 (0.81–1.12)	0.14	0.69 (0.43–1.13)	0.23	1.11 (0.94–1.31)	0.95	1.00 (0.86–1.15)
Colorectal cancer	2774/2102	0.45	1.14 (0.81–1.59)	0.49	0.75 (0.32–1.72)	0.58	0.92 (0.69–1.23)	0.39	1.15 (0.84–1.56)
Liver cancer	1129/1533	*0.04*	0.76 (0.58–0.99)	0.006	1.43 (1.11–1.85)	0.51	1.15 (0.76–1.73)	< 0.0001	0.78 (0.69–0.88)
rs733618									
Overall	3466/5781	0.10	0.81 (0.64–1.04)	0.12	0.91 (0.80–1.02)	0.04	1.27 (1.01–1.59)	0.16	0.88 (0.74–1.05)
Gastric cancer	1095/2509	0.13	0.65 (0.37–1.14)	0.23	0.88 (0.72–1.08)	0.08	1.60 (0.95–2.70)	0.18	0.77 (0.52–1.13)
Colorectal cancer	1089/1489	0.89	1.01 (0.85–1.20)	0.87	0.98 (0.79–1.22)	0.99	1.00 (0.85–1.18)	0.05	0.89 (0.79–1.00)
Liver cancer	671/1126	0.50	0.70 (0.25–1.96)	0.18	0.83 (0.62–1.09)	0.38	1.50 (0.60–3.73)	0.51	0.73 (0.29–1.85)
rs16840252									
Overall	2364/4296	0.64	1.05 (0.85–1.31)	0.94	0.99 (0.73–1.34)	0.44	0.91 (0.72–1.16)	0.89	1.01 (0.83–1.24)
Gastric cancer	809/2075	0.84	0.98 (0.81–1.19)	0.19	1.22 (0.90–1.66)	0.89	0.97 (0.68–1.40)	0.39	0.93 (0.78–1.10)

*Abbreviations*: *OR* odds ratio, *CI* confidence interval, *NA* not available

### Sensitivity analyses

Stabilities of pooled meta-analysis results were examined by omitting one study each time and pooling the results of the other studies. The trends of associations remained unchanged in sensitivity analyses, indicating that our pooled meta-analysis results were statistically stable.

### Publication biases

Publication biases were examined by funnel plots. Funnel plots were overall symmetrical, suggesting that our pooled meta-analysis results were not likely to be severely influenced by publication biases.

## Discussion

CTLA-4 is expressed on activated T cells, and it negatively regulates T cell activation and proliferation. Previous studies have demonstrated that CTLA-4 modulates the duration and strength of T cell-mediated immune responses by competitive binding with co-stimulating B7 molecules and activating of FAS-dependent apoptosis of T cells [9, 10]. Recently, abnormal expression of *CTLA-4* gene has been documented in many types of cancers, and it might contribute to cancer initiation and progression [13–15]. Considering that genetic polymorphisms might influence gene expression or even protein function, *CTLA-4* gene polymorphisms have also been extensively explored with regard to their relationships with different types of malignant disorders. In this meta-analysis, we summarized potential relationships between *CTLA-4* gene polymorphisms and digestive system malignancies by pooling the results of 31 related studies. Our pooled meta-analysis results demonstrated that rs231775 polymorphism was associated with predisposition to colorectal cancer and pancreatic cancer, rs4553808 and rs5742909 polymorphisms were associated with predisposition to gastric cancer, rs3087243 polymorphism was associated with predisposition to liver cancer, and rs733618 polymorphism was associated with predisposition to colorectal cancer. The trends of associations remained unchanged in sensitivity analyses, suggesting that our pooled meta-analysis results were quite statistically stable.

A few points should be considered when interpreting our findings. First, previous experimental studies demonstrated that all investigated polymorphisms might result in altered gene expression or protein structure of CTLA-4 [16, 17]. Thus, it is likely that these polymorphisms might also influence normal functioning of *CTLA-4*, give rise to immune dysfunction, jeopardize anti-tumor immune responses, and influence predisposition to malignancies, and this is the reason why we investigated these polymorphisms in this meta-analysis. Second, although we pooled the results of related studies, the sample size of many comparisons were still relatively small, so future genetic association studies with larger sample sizes are still needed to estimate the relationship between *CTLA-4* polymorphisms and different types of digestive system malignancies so as to get more statistically robust findings. Third, the etiologies and pathogenesis mechanisms of digestive system malignancies are extremely sophisticated, so further association studies also need to investigate the potential influence of gene-gene or gene-environmental interactions on predisposition to digestive system malignancies [18]. Fourth, we aimed to investigate all *CTLA-4* polymorphisms at the beginning. However, we did not find sufficient eligible articles to support pooled meta-analyses of other *CTLA-4* polymorphisms, so we only examined six polymorphisms in this meta-analysis.

Like all meta-analyses, a few limitations of our pooled meta-analyses should also be acknowledged. Firstly, our pooled meta-analysis results were derived from pooling unadjusted findings because we did not have access to raw data of eligible studies [19]. Secondly, environmental factors might also influence relationship between *CTLA-4* polymorphisms and digestive system malignancies. However, most investigators only focused on genetic associations in their works, so gene-environmental interactions were not explored in this meta-analysis [20]. Thirdly, we did not search for grey literatures. Therefore, despite that funnel plots of pooled analyses were overall symmetrical, potential publication biases still might influence the robustness of our pooled results [21].

## Conclusion

So to conclude, this meta-analysis demonstrated that rs231775 polymorphism was associated with predisposition to colorectal cancer and pancreatic cancer, rs4553808 and rs5742909 polymorphisms were associated with predisposition to gastric cancer, rs3087243 polymorphism was associated with predisposition to liver cancer, and rs733618 polymorphism was associated with predisposition to colorectal cancer. These results indicated that these *CTLA-4* polymorphisms might have the potential to serve as genetic biomarkers of digestive system malignancies. Nevertheless, detailed functional analyses are still required to reveal the precise molecular mechanisms of the observed significant associations between *CTLA-4* polymorphisms and digestive system malignancies. Moreover, future studies should also test whether these *CTLA-4* polymorphisms can be used to detect digestive system malignancies in clinical practice.

## Data Availability

The current study was based on the results of relevant published studies.

## Abbreviations

CI: Confidence intervals
CTLA-4: Cytotoxic T lymphocyte-associated antigen 4
HWE: Hardy-Weinberg equilibrium
NOS: Newcastle-Ottawa scale
OR: Odds ratios
